# Molecular Characterization of Endoplasmic Reticulum (ER) Stress-Associated *BiP*, *IRE1*, and *XBP1* Genes in *Diaphorina citri* and Their Roles During *Candidatus* Liberibacter asiaticus Infection

**DOI:** 10.3390/insects17030260

**Published:** 2026-02-28

**Authors:** Zhiyou Xuan, Xinying Yang, Tao Peng, Yingzhe Yuan, Caifu Liu, Yali Wang, Aijun Huang, Long Yi, Xuefeng Wang, Mengji Cao, Changyong Zhou

**Affiliations:** 1 National Citrus Engineering Research Center, Citrus Research Institute, Southwest University, Chongqing 400712, China; xuanzhiyou@email.swu.edu.cn (Z.X.); swuyangxinying@126.com (X.Y.); yyz9498@email.swu.edu.cn (Y.Y.); liucaifu2@163.com (C.L.); wyl1826689226@163.com (Y.W.); wangxuefeng@cric.cn (X.W.); 2 College of Modern Agriculture, Yulin College, Yulin 719000, China; ptky2017@sina.com; 3 National Navel Orange Engineering Research Center, Gannan Normal University, Ganzhou 341000, China; huangaijun@gnnu.edu.cn (A.H.); yilongswu@163.com (L.Y.)

**Keywords:** endoplasmic reticulum (ER) stress, unfolded protein response (UPR), citrus huanglongbing, *Candidatus* Liberibacter asiaticus, *Diaphorina citri*, molecular characterization

## Abstract

The endoplasmic reticulum (ER) stress response, or unfolded protein response (UPR), is crucial for cellular homeostasis and host defense. Its role in insect vectors of plant pathogens remains poorly understood. This study conducted a comprehensive molecular characterization of three core UPR genes—*BiP*, *IRE1*, and *XBP1*—in *Diaphorina citri*, the insect vector of the citrus huanglongbing pathogen *Candidatus* Liberibacter asiaticus (*C*Las). Expression profiling showed distinct developmental and tissue-specific patterns for these genes. The IRE1-mediated unconventional splicing of *XBP1* mRNA was identified in *D. citri* and predicted across diverse insect pests. A semi-quantitative RT-PCR assay was established to detect this splicing event for monitoring ER stress. Time-course analysis of *C*Las-infected *D. citri* revealed an early activation (upregulation of *BiP*, *IRE1*, *XBP1* transcripts, and increased *XBP1* splicing) followed by later suppression of the UPR. Functional studies demonstrated that induction of ER stress with thapsigargin increased *C*Las titer. RNAi-mediated silencing showed that *IRE1* silencing increased *C*Las proliferation, whereas *XBP1* silencing reduced it. These findings provide foundational insights into the ER stress pathway in *D. citri* and demonstrate that the IRE1-XBP1 branch of the UPR plays a critical role in modulating *C*Las infection dynamics within its insect vector.

## Simple Summary

Although endoplasmic reticulum (ER) stress and the consequent unfolded protein response (UPR) are well characterized in mammalian systems, their role in plant pathogens transmitted by insect vectors remains poorly understood. In insects, ER stress genes are not well characterized, leading to a shortage of reliable markers or convenient methods to assess ER stress. Furthermore, the interaction between the citrus huanglongbing pathogen, *Candidatus* Liberibacter asiaticus (*C*Las), and its insect vector, *Diaphorina citri*, is unclear. In this study, we characterized three core UPR genes in *D. citri* and established conserved IRE1-mediated splicing of *XBP1* mRNA as a reliable indicator of ER stress. During *C*Las infection, the UPR was dynamically regulated, showing early activation followed by later suppression. Functional assays demonstrated that ER stress induction increased *C*Las titers, while RNAi silencing revealed that IRE1 and XBP1 have different effects on bacterial proliferation. These findings elucidate a critical host–pathogen interaction and identify potential targets for disease management.

## 1. Introduction

The endoplasmic reticulum (ER) is the largest organelle ubiquitous in eukaryotic cells. It comprises an interconnected network of membrane tubules and sheets and forms specialized membrane contact sites with other organelles. The ER serves as a central hub for protein synthesis, folding, modification, and maturation. Concurrently, it is critically involved in a multitude of other essential biological processes, including lipid synthesis, calcium ion storage and homeostasis, and intracellular signal transduction [1,2,3,4]. When cells are exposed to physiological or pathological stressors, the ER function is disturbed, resulting in the accumulation of unfolded or misfolded proteins within the ER lumen, a condition termed ER stress. In response, cells activate the unfolded protein response (UPR), which modulates a series of processes, including the transcription of relevant genes, mRNA translation, and protein processing and degradation to alleviate ER pressure and restore homeostasis [5,6,7].

The UPR pathway comprises three principal branches mediated by the ER transmembrane protein sensors: inositol-requiring enzyme 1 (IRE1), double-stranded RNA-activated protein kinase (PKR)–like ER kinase (PERK), and activating transcription factor 6 (ATF6) [8,9,10,11]. Under normal conditions, the ER chaperone binding immunoglobulin protein (BiP), also known as glucose-regulated protein 78 (GRP78) or heat shock protein 70 cognate 3 (HSC70), binds to luminal domains of IRE1, PERK, and ATF6, maintaining them in an inactive state. Upon ER stress, the BiP is released to bind misfolded proteins, leading to the oligomerization and phosphorylation of IRE1 and PERK, as well as the cleavage of ATF6. These activated proteins then regulate downstream responses to alleviate stress [5,12,13,14,15]. Among the three branches, the IRE1 cascade is the most conserved. Phosphorylated IRE1 splices the mRNA encoding X-box binding protein-1 (XBP1), resulting in a frameshift that produces the active spliced isoform of the transcription factor XBP1-S. The XBP1-S upregulates genes that facilitate ER protein translocation, folding, and secretion, and those mediating the degradation of misfolded proteins [16,17]. Collectively, the expression levels or activation states of the UPR pathway components serve as specific molecular indicators of ER stress [18,19].

Beyond its role in restoring ER homeostasis, the UPR also plays a crucial role in immunity. Many intracellular pathogens hijack ER nutrients, membrane systems, and secretory functions to fuel their proliferation. Consequently, ER stress and the UPR serve as critical regulatory nexuses in the host-pathogen interplay [20,21,22]. The *Brucella* effector VceC binds to BiP, which induces the UPR, and the RNAi screen reveals that IRE1 was required to facilitate *Brucella* replication in host cells [23,24]. *Listeria* has also been reported to induce ER expansion and UPR. The induction of ER stress by thapsigargin or tunicamycin reduces the intracellular bacterial number [25]. While the *Legionella* suppresses the UPR by inhibiting BiP translation and IRE1-mediated splicing of *XBP1* mRNA [26,27]. Overall, the role of ER stress and the UPR in host–pathogen interactions is complex; however, these mechanisms remain poorly studied in insect systems.

The Asian citrus psyllid (*Diaphorina citri* Kuwayama, Hemiptera: Psyllidae) is the primary insect vector of *Candidatus* Liberibacter asiaticus (*C*Las), which causes the devastating citrus Huanglongbing (HLB, citrus greening) disease around the world [28]. Previous studies have demonstrated that *C*Las exploits the clathrin-mediated endocytosis and the cytoskeletal system of *D. citri* to facilitate its systemic infection and persistent spread [29,30,31]. Transmission electron microscopy observations revealed that *C*Las remodels the ER and induces the formation of ER-associated vacuoles. The bacterium was observed to replicate within these Liberibacter-containing vacuoles (LCVs), similar to the replicative vacuoles formed by other intracellular bacteria like *Brucella* and *Legionella* [32,33,34,35,36]. This suggests a potential role for ER stress in the interplay between *C*Las and *D*. *citri*. *Candidatus* Liberibacter solanacearum (*C*Lso), a closely related bacterium of *C*Las, has been reported to induce ER stress genes and free cytosolic Ca^2+^ in its psyllid vector [37,38]. However, whether *C*Las regulates ER stress in *D. citri*, and the function of the UPR pathway and its related genes during *C*Las infection, remains to be further elucidated.

In this study, we performed a comprehensive characterization of three ER stress-associated genes (*BiP*, *IRE1*, and *XBP1*) and analyzed their expression levels across different developmental stages and tissues in *D. citri*. The unconventional IRE1-mediated splicing of *XBP1* mRNA was identified in *D. citri*, and its occurrence in other insect species was predicted. Accordingly, a semi-quantitative RT-PCR method was developed to detect *XBP1* splicing for monitoring ER stress in *D. citri*. The results indicate that ER stress is induced at the early stage of *C*Las infection but is suppressed at later stages. Induction of ER stress through thapsigargin increased *C*Las titers in *D. citri*. RNA interference revealed that silencing of *IRE1* increased *C*Las titers, whereas silencing of *XBP1* decreased them. These findings contribute to the research on ER stress in insects and provide novel insights into the dynamic host-pathogen interplay underlying *C*Las replication in *D. citri*.

## 2. Materials and Methods

### 2.1. Insects, Plants, and CLas Acquisition

The *C*Las-free *D. citri* population was reared and maintained on orange jessamine (*Murraya koenigii* exLinn.) at the Navel Orange Engineering Research Center of Gannan Normal University in Ganzhou City, Jiangxi Province, China. The insects and plants were kept in insect-proof cages under controlled conditions: temperature 26 ± 1 °C, relative humidity 70 ± 5%, and a photoperiod of 16-h light/8-h dark.

*C*Las-infected *D. citri* adults were obtained as previously described [31]. In brief, fifth-instar nymphs of the *C*Las-free colony were transferred onto *C*Las-infected shoots of Newhall navel orange, which were collected from infected trees near the Tandong citrus orchard at Ganzhou City, Jiangxi Province. As a control, nymphs were similarly placed on healthy Newhall navel orange shoots collected from uninfected trees. All collected shoots were confirmed to be *C*Las-positive or *C*Las-negative by quantitative real-time polymerase chain reaction (qPCR) prior to use for insect feeding. The emerged adults were tested by qPCR after feeding on the shoots. Our experiments, in line with previous reports, confirm that the *C*Las acquisition rate exceeds 95% [31].

### 2.2. RNA Isolation, Complementary DNA Synthesis, qPCR Detection, and Molecular Cloning

Total RNA was extracted using TRIzol reagent (Invitrogen, Carlsbad, CA, USA, 15596018CN) according to the manufacturer’s instructions. RNA concentration and purity were assessed using a NanoDrop spectrophotometer (Thermo Fisher Scientific, Waltham, MA, USA). For qPCR detection, 0.5 μg of total RNA was reverse-transcribed using the NovoScript^®^ Plus All-in-one 1st Strand cDNA Synthesis SuperMix (Novoprotein, Suzhou, China, E047). Quantitative PCR (qPCR) was performed with the NovoStart^®^ SYBR qPCR SuperMix Plus (Novoprotein, Suzhou, China, E096) on a LightCycler^®^ 480 System (Roche Diagnostics, Mannheim, Germany). The reaction procedures were as follows: preincubation at 95 °C for 30 s followed by 40 cycles of amplification at 95 °C for 10 s, 60 °C for 20 s, and 72 °C for 20 s. The *DcGAPDH* gene was used as an internal reference. The relative expression level of target genes was calculated using the 2^−ΔΔCT^ method.

For molecular cloning, 1 μg of total RNA was reverse-transcribed using the Reverse Transcriptase M-MLV (TaKaRa Bio USA, Inc., San Jose, CA, USA, 2641A). PCR amplification was subsequently performed using PrimeSTAR^®^ Max DNA Polymerase (TaKaRa Bio USA, Inc., San Jose, CA, USA, R045Q) with gene-specific primers (Table S1). Rapid Amplification of cDNA Ends (RACE) was conducted using specific primers along with 5′ or 3′ adaptors. The amplification products were separated by gel electrophoresis, purified using the TIANgel Purification Kit (TIANGEN, Beijing, China, DP219), and cloned with the pEASY^®^-Blunt Cloning Kit (TransGen Biotech, Beijing, China, CB101-01). Finally, the cloned fragments were sent to BGI (Beijing Genomics Institute, Shenzhen, China) for sequencing.

### 2.3. Bioinformatic and Statistical Analysis

To identify endoplasmic reticulum (ER) stress-associated genes, we conducted gene annotation searches and homology analyses using publicly available *D. citri* genome and transcriptome data from the China National GeneBank DataBase (accession: CNP0003364) [39], and the NCBI GeneBank database (accession: NW_007377701.1). The retrieved sequences served as references for subsequent experimental validation. Validated sequences were then re-mapped to the *D. citri* genome (CNP0003364) to determine their precise chromosomal locations and exon-intron organizations.

Homology sequence alignments and open reading frame (ORF) predictions were performed using CLC Genomics Workbench 11. Conserved protein domains were analyzed via the Conserved Domain Search Service (CD Search) on NCBI. Tertiary protein structures were predicted using AlphaFold 3 and visualized with PyMOL 3.1.3. Phylogenetic analysis was conducted by constructing a maximum-likelihood tree in MEGA 7 software. The physicochemical properties of the proteins were predicted using the ExPASy ProtParam tool. Statistical analyses were performed using unpaired *t*-tests or one-way ANOVA followed by Tukey’s HSD test in GraphPad Prism 10.1.2, and results were visualized accordingly. All figures were assembled and edited using Adobe Illustrator CC 2017.

### 2.4. Semi-Quantitative RT-PCR for Detecting XBP1 Splicing

Semi-quantitative RT-PCR was performed to analyze *XBP1* splicing. cDNA synthesis was conducted as described above. PCR amplification was carried out using a Green Taq Mix (Vazyme, Nanjing, China, P131-02) with *XBP1*-specific primers (Table S1) spanning the spliced intron. The *GAPDH* gene was amplified in parallel as an internal control. Each 20-μL PCR reaction contained 1 μL of cDNA template (equivalent to ~16 ng RNA), 0.5 μL each of forward and reverse primers (10 μM), 10 μL of Green Taq Mix, and 8 μL of ddH_2_O. The PCR program was set as follows: initial denaturation at 95 °C for 3 min followed by 35 cycles (for *XBP1*) or 30 cycles (for *GAPDH*) of denaturation at 95 °C for 15 s, annealing at 56 °C (*XBP1*) or 60 °C (*GAPDH*) for 15 s, and extension at 72 °C for 25 s, with a final extension at 72 °C for 5 min.

To clearly distinguish the band of *XBP1-S* and *XBP1-U*, 13 μL of the PCR product was mixed with 1 μL of FastDigest EcoRI (Thermo Fisher Scientific, Waltham, MA, USA, FD0274) and 1 μL of 10× FastDigest Green Buffer. The 15 μL digestion mixture was incubated at 37 °C for 30 min. The digested products were then separated by agarose gel electrophoresis. Band intensities (gray values) were quantified using ImageJ 1.53k. The relative expression level of spliced *XBP1-S* for each sample was calculated using the formula: (*XBP1-S* gray value/*GAPDH* gray value of the sample)/(average *XBP1-S* gray value/*GAPDH* gray value of the control group).

### 2.5. Thapsigargin Treatment, dsRNA Synthesis, and Injection

To assess the effect of endoplasmic reticulum (ER) stress on *C*Las, the ER stress inducer Thapsigargin (Beyotime, Shanghai, China, SC0389-2 mM) was used. The stock solution was diluted with ddH_2_O to a working concentration of 20 µM. Adults that had emerged after feeding for one week on the same *C*Las-infected branches were microinjected with 40 nL of either the 20 µM Thapsigargin solution or a 1% DMSO solution (control). Mortality was recorded 2 days post-injection. Surviving insects were then collected for subsequent determination of *C*Las titers.

To evaluate the influence of ER stress-related genes on *C*Las, double-stranded RNA (dsRNA) targeting specific genes was synthesized in vitro for RNA interference (RNAi). For each target gene, a fragment was amplified by PCR using gene-specific primers containing the T7 promoter sequence at their 5′ ends. The PCR product served as the template for dsRNA synthesis, using the TranscriptAid T7 High Yield Transcription Kit (Thermo Fisher Scientific, Waltham, MA, USA, K0441) according to the manufacturer’s protocol. dsRNA targeting the green fluorescent protein (GFP) gene was synthesized in parallel as a negative control. The synthesized dsRNA was diluted to a working concentration of 2000 ng/µL. *C*Las infected insects were microinjected with 40 nL of the corresponding dsRNA solution. Mortality was recorded at 4 days post-injection, and surviving insects were collected for subsequent quantification of *C*Las titers.

### 2.6. DNA Extraction and Quantitative Detection of CLas Titer

Total DNA was extracted using a modified CTAB method. Briefly, individual insects or 100 mg of plant leaf tissue were flash-frozen and ground in a microcentrifuge tube. Then, 500 µL (for insects) or 800 µL (for plant tissue) of CTAB extraction buffer was added, followed by incubation at 65 °C for 15 min. An equal volume of DNA Extraction Reagent 25:24:1 (G-CLONE, Beijing, China, EX0128) was added, and the mixture was vortexed thoroughly, incubated at room temperature for 10 min, and centrifuged at 12,000 rpm for 10 min. The aqueous phase was transferred to a new tube, mixed with an equal volume of Nucleotide Extraction Reagent 24:1 (G-CLONE, EX0100), vortexed, incubated for 5 min, and centrifuged at 12,000 rpm for 5 min. The supernatant was collected, and DNA was precipitated with an equal volume of isopropanol (Macklin, Shanghai, China, I811925). The pellet was washed twice with 75% ethanol, air-dried, and finally dissolved with an appropriate amount of ddH_2_O for subsequent analysis.

The *C*Las titer was quantified by a probe-based quantitative PCR (qPCR) method as previously reported [40]. Briefly, total DNA extracted from individual *D. citri* was diluted to a concentration of 100 ng/µL. Each 20 µL qPCR reaction contained 10 µL of Premix Ex Taq (Probe qPCR) (TaKaRa Bio USA, Inc., San Jose, CA, USA, RR390A), 0.3 µL of probe, 0.4 µL each of the primers HLBr and HLB4G, and 1 µL of template DNA (100 ng). Reactions were run on a LightCycler^®^ 96 system (Roche Diagnostics, Mannheim, Germany.) under the following conditions: 95 °C for 3 min; 40 cycles of 95 °C for 10 s and 60 °C for 30 s. The absolute quantification of *C*Las copy number was calculated using the standard curve equation y = −4.11x + 55.508 (R^2^ = 0.9964), where y represents the Ct value and x represents the log10-transformed copy number. The *C*Las titer was expressed as 10^x^ copies per 100 ng of total *D. citri* DNA.

## 3. Results

### 3.1. Identification and Sequence Analysis of BiP, IRE1 Genes in Diaphorina citri

**Figure 1:**
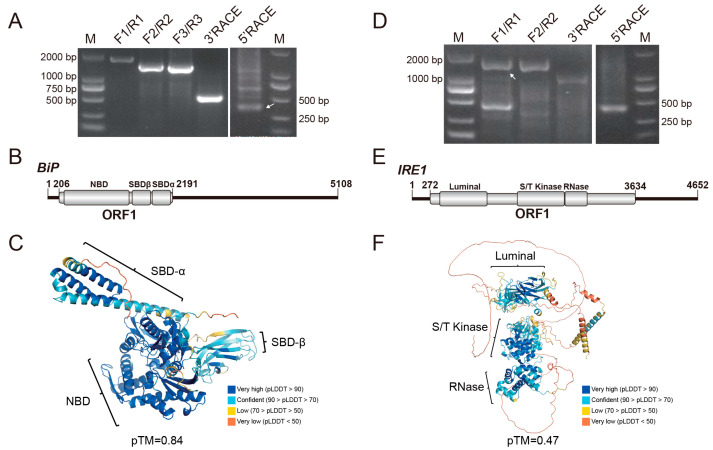
Amplification and sequence analysis of *BiP* and *IRE1*. (**A**,**D**) Full-length amplification of *BiP* and *IRE1* transcripts, respectively. Primers used are listed in Table S1. Lane M: DL2000 DNA marker. White arrowheads indicate the specific amplicons. (**B**,**E**) Molecular organizations of *BiP* and *IRE1* transcripts, respectively. Open reading frames (ORFs) and conserved domains are denoted as gray boxes. (**C**,**F**) Predicted three-dimensional structures of the BiP and IRE1 proteins, respectively. Conserved domains are highlighted, and model quality scores (pLDDT: predicted Local Distance Difference Test; pTM: predicted Template Modeling score) are provided.

Using RT-PCR and RACE amplification, the full-length transcript sequence of the *DcBiP* gene was obtained (Figure 1A). Genomic localization revealed that the *DcBiP* gene is located on chromosome 10, where it occupies approximately 17.5 kb and comprises 12 exons (Table S2). The full-length *BiP* mRNA is 5108 nt, with a 5′ UTR of 205 nt, a 3′ UTR of 2917 nt, and an ORF of 1986 nt that encodes a protein of 661 amino acids. The BiP protein of *D. citri*, with a molecular weight of 72.79 kDa, contains a nucleotide-binding domain (NBD) and a substrate-binding domain (SBD) (Figure 1B). Consistent with known structures [41,42], the predicted three-dimensional (3D) model (pTM = 0.84) of BiP displays a characteristic bilobed architecture for the NBD and a β-sandwich fold with an α-helical lid for the SBD (Figure 1C). Multiple sequence alignment demonstrated that BiP is highly conserved across mammals and insects; the most variable regions were localized to the N-terminal segment of the signal peptide and the C-terminal region preceding the conserved KDEL endoplasmic-reticulum retrieval signal (Figure S1). The overall pairwise sequence identities of BiP protein across mammals and insects are 78–100%, the BiP of the potato psyllid (*Bactericera cockerelli*) showed the highest sequence identities (95.59%) with *D. citri* (Table S3), phylogenetic tree also grouped them together with other hemipteran insects (Figure S2A), suggest the closed evolution relationship between *B. cockerelli* and *D. citri*.

The *DcIRE1* gene maps to chromosome 7, spans approximately 29.6 kb, and contains 17 exons. The full-length mRNA of *IRE1* was verified to be 4652 nt (Figure 1D), comprising a 271 nt 5′ UTR, a 1018 nt 3′ UTR, and a 3363 nt ORF that encodes a 1120 aa protein with a molecular weight of 125.56 kDa (Table S2). Conserved domain search identified the characteristic luminal, Serine/Threonine kinase, and the RNase domain of IRE1 protein (Figure 1E). Multiple sequence alignment indicated that these domains are more conserved than other regions. In addition, the conventional phosphorylated serine was identified as the position 632 amino acid in the S/T kinase domain and was shown to be highly conserved across mammals and insects (Figure S3). The overall predicted 3D model showed a relatively low pTM score (0.47), while the conserved regions displayed high confidence (pLDDT > 70), supporting the reliability of the core fold (Figure 1F). Sequence identities of IRE1 among different insect orders ranged between 40% and 60% (Table S4). In the phylogenetic tree, *D. citri* IRE1 clustered with other hemipteran representatives and was most closely related to the IRE1 of *Bemisia tabaci* (Figure S2B).

### 3.2. Identification of XBP1 Gene and Its Unconventional mRNA Splicing

Although the protein domains of BiP and IRE1 are conserved, commercial antibodies were unsuccessful in stably detecting their protein level to monitor ER stress in *D. citri*. We also developed specific antibodies against IRE1 peptides around phosphorylated Ser 632, and it too proved ineffective. Therefore, we tried to identify the *XBP1* gene in *D. citri*, as the IRE1-mediated unconventional splicing of *XBP1* mRNA is a conserved and widely used molecular indicator for monitoring ER stress in metazoans [18,19].

The full-length cDNA sequence was obtained first by RT-PCR and RACE (Figure S4A). To determine the precise IRE1-spliced site of *XBP1* mRNA, we compared previously verified *XBP1* splicing sites in other species and predicted that it was located downstream of the bZIP_XBP1 domain, adjacent to the conserved CNG’NNG sequence. Then, the specific primers flanking the predicted intron were designed to simultaneously amplify the spliced and unspliced isoform (*XBP1-S*/*U*). However, due to the short length of the excised intron, the two isoforms could not be resolved by standard agarose gel electrophoresis, so we utilized an EcoRI restriction site within the intron to digest the *XBP1-U* into two smaller bands of the same size (Figure S4B). The undigested *XBP1-S* band was cloned and subjected to Sanger sequencing to confirm the splice junction location.

**Figure 2:**
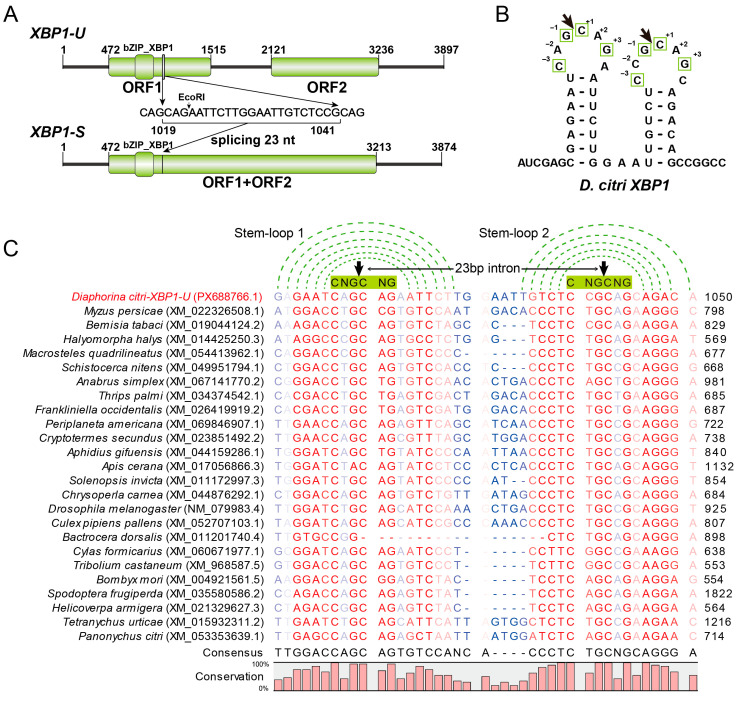
Sequence analysis of *XBP1*. (**A**) Molecular organizations of the unspliced *XBP1-U* and spliced *XBP1-S* transcripts. Open reading frames (ORFs) and conserved domains are denoted by green boxes. The sequence of the 23-nt intron removed by splicing is shown, with the EcoRI recognition site highlighted. (**B**) Predicted secondary stem-loop structure of the region surrounding the splice site in *D. citri XBP1* mRNA. The arrow indicates the cleavage site, and conserved nucleotides critical for cleavage are boxed. (**C**) Multiple sequence alignment of the splice site regions from *D. citri XBP1* and its predicted orthologs from other pests. The conserved stem structure is indicated by green dotted lines connecting base-paired nucleotides. The six-nucleotide CNG’CNG recognition sequence in the loop is highlighted with a green box. The cleavage site is marked by a black arrowhead. Sequence conservation is visualized by color intensity (red to blue scale, from high to low conservation).

The spliced intron of *XBP1-U* mRNA was confirmed to be 23 nt (positions 1019–1041). The full-length *XBP1-U* transcript is 3897 nt, while the spliced *XBP1-S* is 3874 nt (Figure 2A). The corresponding *DcXBP1* gene was mapped to chromosome 8 and spans approximately 14.8 kb (Table S2). The *XBP1-U* transcript comprises five exons and contains two physically separated open reading frames (ORFs). ORF1 encodes a 347 aa protein that contains a conserved bZIP domain that was designated XBP1-U. ORF2 is located 615 nt downstream of ORF1 and would not be expressed without IRE1-mediated splicing (Figure 2A). The splicing event induces a frameshift, resulting in an *XBP1-S* mRNA that harbors a novel, continuous ORF that fuses ORF1, the interval 615 nt sequence, and ORF2. This composite ORF encodes a 939 aa protein, identified as the functional XBP1-S.

The predicted 3D structures of XBP1-U and XBP1-S proteins comprise multiple α-helices, including the basic leucine zipper (bZIP) domains with high confidence (pLDDT > 90) (Figure S5A). Both isoforms contained relatively long intrinsically disordered regions, which correlate with their low structural stability and transcription factor roles [16,43]. On the sequence level, XBP1 exhibits low sequence identities across different insect orders, ranging from approximately 20% to 40% (Table S5). Sequence alignment revealed that only the bZIP domain could be reliably aligned, within which the basic region residues are relatively conserved (Figure S5B). The phylogenetic analysis reveals that hemipteran XBP1 sequences are non-monophyletic. One subgroup, comprising *Halyomorpha halys* and *Myzus persicae*, clusters with mammals, while another subgroup, containing *D. citri*, *B. tabaci*, and *Macrosteles quadrilineatus*, forms a separate clade with other insect orders (Figure S4B).

Further analysis of the spliced intron revealed that both the 5′ and 3′ splice sites can form two characteristic stem-loop structures with the conserved CNG′CNG motifs located within the loop regions (Figure 2B). Since the splicing sites of *XBP1* have not been identified in most insect pests, we extended our analysis by manually blasting and identifying these sites in representative pest species. Sequence alignment (Figure 2C) showed that the spliced intron length is 23 nt in most species, while in members of Lepidoptera and Coleoptera, it is 17 nt. Importantly, the stem-loop structures and the CNG′CNG cleavage motif remain highly conserved across these examined species, suggesting the splicing mechanism of *XBP1* is evolutionarily conserved among diverse insect pests.

### 3.3. Establishment of a Semi-Quantitative RT-PCR Method for Detecting XBP1 Splicing to Evaluate ER Stress

To quantify *XBP1* splicing, we first designed two qPCR primers: one spanning the intron to detect *XBP1-S*, and the other targeting the 3′ region within the intron to detect *XBP1-U*. Due to the presence of multiple CAG repeats near the splicing site (Figure S6A), which may cause nonspecific amplification, we evaluated primer specificity by performing RT-PCR using plasmids containing *XBP1-U* or *XBP1-S* individually. Primers capable of detecting total *XBP1* (*XBP1-T*, comprising both *XBP1-U* and *XBP1-S*) were used as a control. The results revealed that both primer sets produced nonspecific amplification products (Figure S6B). To further investigate whether nonspecific amplification persists in the presence of both templates, we used a plasmid containing a longer *XBP1-U* fragment and mixed it with the *XBP1-S* plasmid at varying ratios before amplification. Nonspecific amplification was still observed under these conditions (Figure S6C). These findings suggest that specific detection of *XBP1-S* is challenging because the primers have been computationally predicted to exhibit relatively high theoretical specificity.

**Figure 3:**
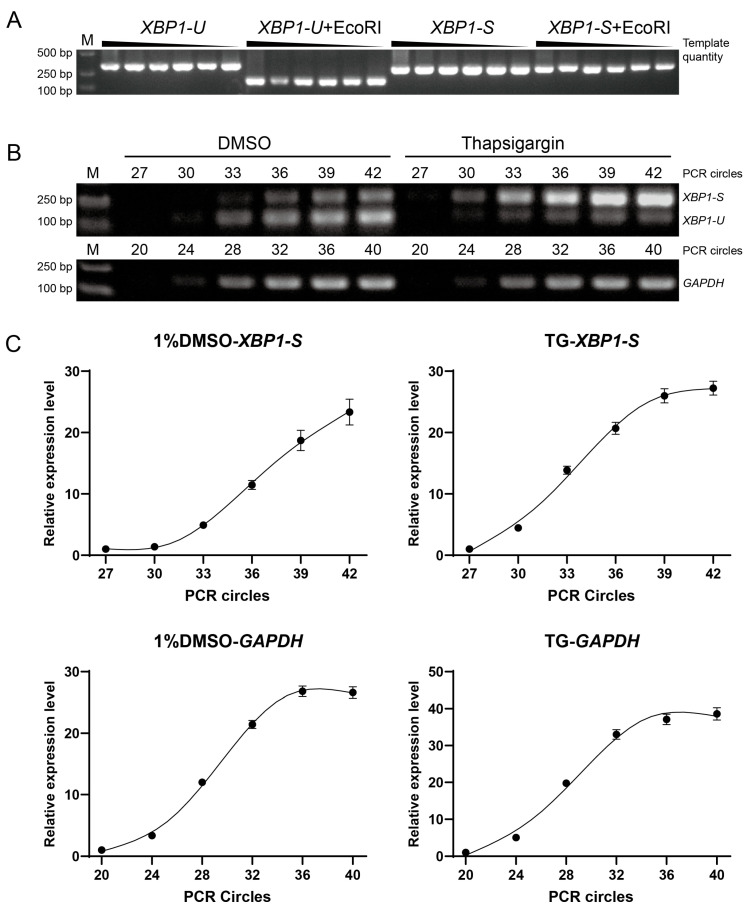
Development of a semi-quantitative RT-PCR method for detecting *XBP1* splicing. (**A**) Specificity validation of the EcoRI cleavage assay. Plasmids containing the unspliced *XBP1-U* or spliced *XBP1-S* fragment were amplified by PCR. Serial dilution of the template showed consistent band intensity, indicating amplification saturation. Subsequent EcoRI digestion specifically and completely cleaved the *XBP1-U*, but not the *XBP1-S*, confirming the assay’s ability to distinguish splicing isoforms. (**B**) Determination of optimal PCR cycle numbers. Amplifications for the reference gene (*GAPDH*) and *XBP1-S* were established using RNA from insects treated with a solvent control (1% DMSO) or an ER stress inducer (20 µM thapsigargin). This aimed to identify a cycle number suitable for detection across varying levels of ER stress. (**C**) Quantification of the amplification with different cycles, as shown in (**B**). The band intensity at cycle 27 (for XBP1-S) and cycle 20 (for GAPDH) was normalized to 1, and the relative expression levels (*Y*-axis) of three replicates were plotted against the PCR cycle number (*X*-axis).

We therefore developed a semi-quantitative RT-PCR assay to detect *XBP1* splicing. To ensure that the band intensity from gel electrophoresis accurately reflects mRNA levels, we first verified that EcoRI completely digests *XBP1-U* even at maximal PCR product yield, while *XBP1-S* remains intact (Figure 3A). Subsequently, we determined the cycle numbers within the linear amplification phase for both *XBP1-S* and the reference gene (*GAPDH*) under normal conditions and during ER stress induced by thapsigargin (Figure 3B). Accordingly, the optimal cycle numbers were determined to be 35 for *XBP1-S* and 30 for *GAPDH* (Figure 3C). Detailed reaction procedures and conditions are described in the Section 2.

### 3.4. Developmental and Tissue Expression Patterns of BiP, IRE1, and XBP1 Genes in D. citri

**Figure 4:**
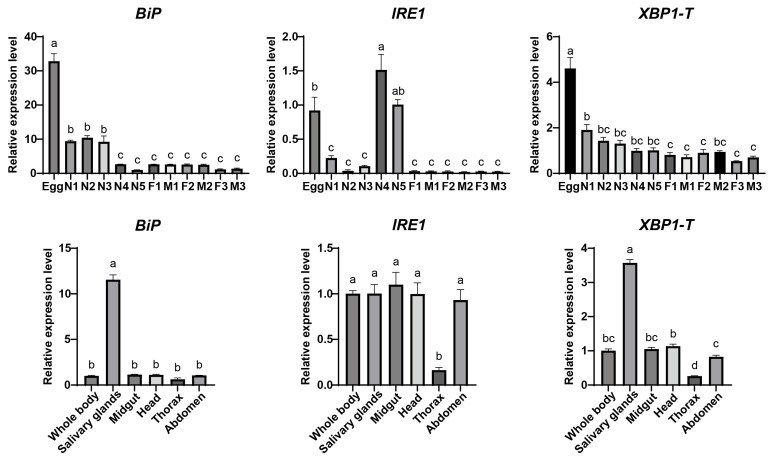
Spatial and temporal expression analysis of *BiP*, *IRE1*, and *XBP1* genes. Relative transcript levels of *BiP*, *IRE1*, and *XBP1-T* (total *XBP1*) were analyzed across developmental stages and different tissues. Developmental stages: first- to fifth-instar nymphs (N1 to N5). 1-, 2-, and 3-day-old females (F1, F2, F3) and males (M1, M2, M3). Data are presented as mean ± SEM. Statistical significance was determined by one-way ANOVA followed by Tukey’s HSD test. Different lowercase letters above bars indicate significant differences (*p* < 0.05).

The expression patterns of the *DcBiP*, *DcIRE1*, and *DcXBP1* genes were analyzed by quantitative real-time PCR (qPCR) (Figure 4). The developmental stages, including eggs, from the 1st to 5th instar nymphs (N1–N5), and from 1- to 3-day-old female and male adults (F/M, 1–3). The results showed that the expression levels of all three genes were relatively low in the adult stage, with no significant differences between sexes. Specifically, *BiP* and *XBP1* exhibited the highest expression during the egg stage, which gradually decreased throughout development. *IRE1* expression peaked in the 4th and 5th instar nymphs, followed by the egg stage. Tissue-specific expression analysis revealed that *BiP* was highly expressed in the salivary gland, with no significant differences in other tissues. Similarly, *XBP1* showed the highest expression in the salivary gland and the lowest in the thorax. *IRE1* expression was lowest in the thorax, while no obvious differences were observed among other tissues.

### 3.5. Temporal Response of the ER Stress Associated BiP, IRE1, and XBP1 Genes in D.citri to CLas Infection

**Figure 5:**
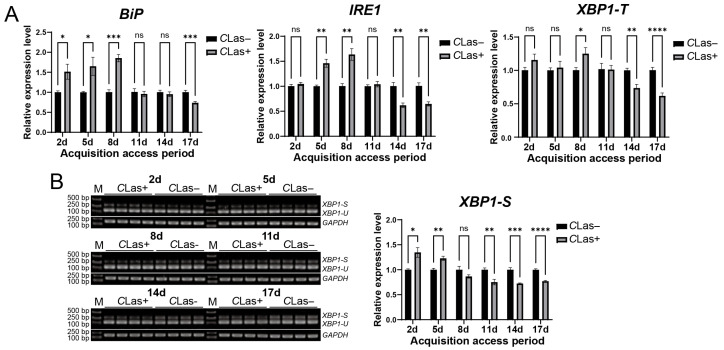
Temporal response of the ER stress-associated *BiP*, *IRE1*, and *XBP1* genes in *D. citri* to *C*Las infection. (**A**) Relative transcript levels of *BiP*, *IRE1*, and *XBP1-T*. Late fifth-instar nymphs were allowed to feed on *C*Las-infected or healthy citrus plants. Gene expression was analyzed in emerged adults at 2, 5, 8, 11, 14, and 17 days after the initiation of feeding. (**B**) Corresponding splicing levels of *XBP1* in emerged adults at the same time points of (**A**). The relative expression level of spliced *XBP1-S* was calculated using the formula: (*XBP1-S* gray value/*GAPDH* gray value of the sample)/(average *XBP1-S* gray value/*GAPDH* gray value of the control group). Data are presented as mean ± SEM. Significant differences between the infected and healthy groups at each time point were determined by an unpaired *t*-test. Asterisks denote statistical significance: *, *p* < 0.05; **, *p* < 0.01; ***, *p* < 0.001; ****, *p* < 0.0001; ns, not significant.

To assess the response of ER stress genes in *D. citri* to *C*Las infection, late fifth-instar nymphs were fed on either healthy or *C*Las-infected citrus branches. Gene expression levels in emerged adults were compared after different feeding durations. Compared with the control group, *BiP* level was significantly upregulated at 2, 5, and 8 days post-feeding (dpf) on *C*Las-infected *D. citri* but was downregulated at 17 dpf. *IRE1* level was upregulated at 5 and 8 dpf and downregulated at 14 and 17 dpf. The total *XBP1* transcript level was elevated at 8 dpf and reduced at 14 and 17 dpf (Figure 5A). Furthermore, semi-quantitative RT-PCR analysis of *XBP1* splicing revealed that the spliced *XBP1-S* was significantly upregulated at 2 and 5 dpf, and downregulated at 11, 14, and 17 dpf (Figure 5B). In summary, although the exact timing of up- and down-regulation varied among these genes, their expression collectively exhibited an early induction followed by later suppression. These results indicate that ER stress is activated during the early phase of *C*Las acquisition in *D. citri* but is suppressed in the later phase.

### 3.6. Effect of ER Stress on CLas Titers in D. citri

**Figure 6:**
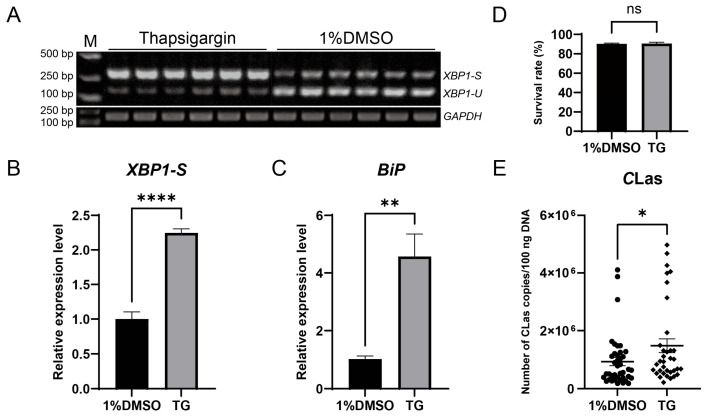
Induction of ER stress by thapsigargin upregulated *C*Las titers in *D. citri*. (**A**) Detection of *XBP1* mRNA splicing in *D. citri* treated with 20 µM thapsigargin (TG) or the solvent control (1% DMSO). (**B**) Quantification of the spliced *XBP1-S* from (**A**). The relative expression level of spliced *XBP1-S* was calculated using the formula: (*XBP1-S* gray value/*GAPDH* gray value of the sample)/(average *XBP1-S* gray value/*GAPDH* gray value of the control group). (**C**) Relative expression level of *BiP* mRNA under the same treatments. (**D**) Survival rate of *D. citri* adults two days after treatment with TG or DMSO. (**E**) *C*Las titers in *D. citri*. Fifth-instar nymphs were first allowed a 7-day acquisition access period on *C*Las-infected citrus shoots, then the emerged adults were treated with 20 µM TG or 1% DMSO, and the bacterial titer was quantified two days post-treatment. Data are presented as mean ± SEM. Significant differences were determined by an unpaired *t*-test. Asterisks denote statistical significance: *, *p* < 0.05; **, *p* < 0.01; ****, *p* < 0.0001; ns, not significant.

**Figure 7:**
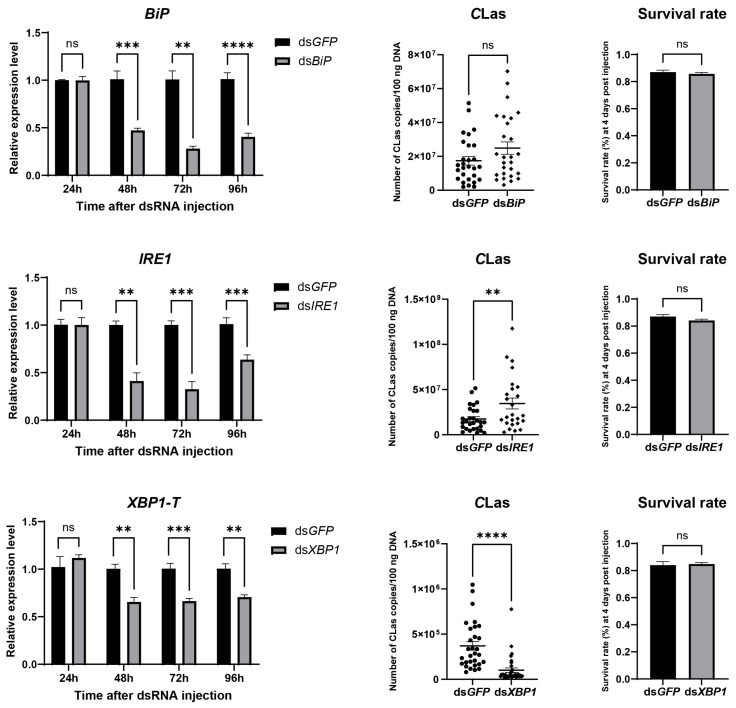
Effect of silencing *BiP*, *IRE1*, and *XBP1* on *C*Las titers in *D. citri*. Insects were injected with dsRNA (40 nL per insect at 2000 ng/µL) targeting *BiP*, *IRE1*, or *XBP1*, using dsRNA targeting *GFP* as a control. For each target gene: (**Left**) panel: Silencing efficiency, presented as the relative mRNA levels of the target gene at different time points post-injection. (**Middle**) panel: *C*Las titers in infected psyllids at 4 days post-injection (dpi). (**Right**) panel: Survival rate of psyllids at 4 dpi. Data are presented as mean ± SEM. Significant differences were determined by an unpaired *t*-test. Asterisks denote statistical significance: **, *p* < 0.01; ***, *p* < 0.001; ****, *p* < 0.0001; ns, not significant.

To evaluate the impact of ER stress on the titer of *C*Las in *D. citri*, *C*Las-infected *D. citri* were injected with the ER stress inducer thapsigargin (20 µM). The result showed successful ER stress induction, confirmed by upregulation of both *BiP* and *XBP1-S*, which led to a significant increase in *C*Las titer without significantly affecting psyllid survival (Figure 6). To further examine the roles of specific ER stress genes in modulating *C*Las titer, *BiP*, *IRE1*, and *XBP1* were individually silenced via dsRNA injection. Each gene was effectively downregulated at 2, 3, and 4 days post-injection, with no significant impact on psyllid survival. Silencing *BiP* resulted in a modest, non-significant rise in *C*Las titer. In contrast, *IRE1* silencing significantly enhanced *C*Las titer, whereas *XBP1* silencing significantly reduced it (Figure 7). These results demonstrate that the ER stress pathway, particularly IRE1 and XBP1, plays critical roles in regulating *C*Las proliferation within its insect vector.

## 4. Discussion

The endoplasmic reticulum (ER) plays a crucial role in maintaining cellular homeostasis. The unfolded protein response (UPR) serves as a key pathway in restoring ER function and activating signaling pathways associated with innate immunity and host defense. While the ER stress and the UPR have been extensively studied in mammals and plants, their role in insects, particularly in agricultural pests, remains less explored. Here, we conduct a comprehensive characterization of three ER stress-associated genes (*BiP*, *IRE1*, and *XBP1*) in *D. citri* (Figure 1 and Figure 2), the insect vector of *C*Las that causes devastating citrus huanglongbing disease in the field. Through sequence alignment, conserved domain analysis, three-dimensional structure prediction, and phylogenetic analysis (Figures S1–S5), we conclusively demonstrate that these three genes are authentic orthologs of the key functional components in the UPR pathway of *D. citri*. What is more, we identified the IRE1-mediated *XBP1* splicing event and predicted its occurrence in other insects (Figure 2). These results, combined with their temporal and spatial expression analysis (Figure 4), provide fundamental information for the study of ER stress in *D. citri* and other insects. Although the splicing and the frame shift mechanism of *XBP1* mRNA are conserved across mammals and insects, the resulting XBP1-S protein in *D. citri* is notably larger than those previously reported. This difference arises because the two ORFs in *D. citri* are separated by a 615 nt interval sequence, rather than overlapping as observed in other species [16,44]. Therefore, this structural distinction may reflect an evolutionary adaptation associated with a distinct functional role for XBP1-S in *D. citri*, that need further experimental validation.

The splicing of *XBP1* mRNA is widely regarded as a stable indicator for monitoring ER stress [18,19]. In this study, we developed a semi-quantitative RT-PCR method to detect *XBP1* splicing in *D. citri*. The clear induction of *XBP1-S* upon treatment with the ER stress inducer thapsigargin further confirms the feasibility of this approach (Figure 3B and Figure 6A). Although real-time qPCR would offer greater precision and convenience, our experimental results indicate that designing specific primers to detect *XBP1-S* in *D. citri* is challenging due to the presence of multiple CAG repeats around the cleavage site (Figure S6). While the qPCR-based methods may still be feasible in other insects, as some insects do not possess such extensive CNG repeats (Figure 2C). Previous studies evaluate qPCR primer specificity using melt curve analysis or amplicon sequencing [45,46]. However, non-specific amplification from *XBP1-U* could yield products that are identical or differ by only about twenty nucleotides from the specific *XBP1-S* product, making it difficult to distinguish them based on a single melt curve peak or by sequencing. Therefore, we recommend assessing primer specificity as shown in Figure S6C, using cDNAs of *XBP1-U* and *XBP1-S* with different lengths, so that the amplicons from different isoforms can be clearly distinguished.

To monitor ER stress in *D. citri* upon *C*Las infection, we conducted a time-course experiment and quantified the transcript levels of *BiP*, *IRE1*, and *XBP1*. Although their protein levels were not assayed due to antibody unavailability, the spliced form of *XBP1* mRNA serves as a well-established indicator of IRE1 kinase/RNase activity, and *BiP* expression is regulated by the transcription factor XBP1-S and ATF6 [47,48], which corresponds to the result that expression of *BiP* transcripts appeared somewhat delayed relative to the *XBP1* splicing (Figure 5). Overall, the results suggest that the UPR, particularly the IRE1-XBP1 branch, is activated in *D. citri* during the early stages of *C*Las infection and becomes suppressed in later stages. A recent study also showed that PERK is activated in the carrot psyllid *Bactericera trigonica* following *C*Lso infection, suggesting that a similar response may occur in *C*Las-*D. citri* system. Moreover, since the *ATF6* gene has not been identified so far, the *C*Las infection triggers a broad UPR response or only affects specific pathways still needs further investigation.

It remains unclear whether the detected expression changes are driven by *C*Las or represent a host adaptive response. Many intracellular pathogens have evolved to manipulate the UPR to promote infection. For instance, *Simkania negevensis* induces ER stress and subsequently downregulates it, and the bacterium can prevent the ER stress induced by tunicamycin or thapsigargin [49]. Dengue virus regulates UPR in a time-dependent manner to prevent apoptosis and prolong life cycle [50]. The prolonged UPR can trigger autophagy and apoptosis. Previous studies have shown that *C*Las infection induces apoptosis and antibacterial autophagy [51,52,53,54]. *Brucella* effectors can induce the UPR and autophagy to promote replication [55,56]. The *C*Las effector SDE4040 has been reported to induce autophagy in *Spodoptera frugiperda* (Sf9) cells [52]. It is possible that *C*Las can exploit effectors to selectively block or stimulate the UPR to modulate host autophagy or apoptosis. Additionally, microRNAs may also be involved in this regulation, as one has been shown to target 3′ UTR of *ATG16L1* to modulate *C*Las-induced autophagy in *D. citri* [54], while our characterization of the precise 5′ and 3′ UTR sequences of the three gene transcripts will contribute to this line of research (Figure 1 and Figure 2).

ER stress exerts pathogen-specific effects during intracellular bacterial infections. The UPR promotes the proliferation of *Brucella* [57,58], but suppresses *Listeria* [25]. In *Chlamydia*, inhibiting the IRE1 and PERK disrupts inclusion formation, whereas UPR-induced inflammation negatively impacts bacterial survival [59,60]. Here, we observed that induction of ER stress by thapsigargin increased the *C*Las titer in *D. citri* (Figure 6). This result is consistent with recent studies showing that thapsigargin treatment elevated *C*Lso titer in *B. trigonica* [37,61] but differs from the finding that thapsigargin reduced *C*Las titer in *D. citri* [61]. This discrepancy may be attributed to the concentration of thapsigargin used: we applied 20 μM, whereas the latter study employed 40 μM. A higher concentration may trigger a more intense ER stress, potentially activating downstream autophagy or apoptosis pathways that could adversely affect bacterial survival.

Return to the data on the expression of UPR-related genes in response to *C*Las infection, assuming that the early upregulation and later downregulation of the UPR result from active manipulation by *C*Las, this pattern suggests that a mild or transient UPR is beneficial for the pathogen, whereas a sustained or strong UPR is likely detrimental. This assumption aligns with the result that thapsigargin-induced UPR promoted *C*Las proliferation. Furthermore, the reduction in *C*Las titer upon *XBP1* silencing suggests that the XBP1-mediated adaptive pathway, which restores ER morphology and promotes cell survival [62,63], likely provides a supportive environment for *C*Las. Conversely, the increase in *C*Las titer following IRE1 silencing implies that IRE1 counteracts infection through other pathways. This protective role may be mediated by its XBP1-independent arms, such as the kinase-activated JNK signaling and the regulated IRE1-dependent decay (RIDD), which can trigger defense responses like apoptosis or antimicrobial autophagy detrimental to the pathogen [64,65,66,67]. Future studies could directly test this speculation using specific inhibitors targeting the kinase or RNase domains of IRE1, assessing its phosphorylation, and analyzing the expression of XBP1 target genes, RIDD markers, and JNK pathway-related genes. We also used several other ER stress and IRE1-specific modulators (tunicamycin, 4-phenylbutyric acid, 4μ8C, STF-083010, IXA4, and sunitinib), but none elicited the expected effects under the tested conditions, and higher concentrations of these compounds often led to increased mortality of *D. citri*. Despite these challenges, the results from thapsigargin-treatment and genetic silencing experiments indicate that a modulated UPR response, particularly through the IRE1-XBP1 cascade, plays a critical role in *C*Las infection dynamics. These findings provide valuable insights into ER stress–*C*Las interactions in *D. citri*, though further evidence is needed to fully elucidate the roles of ER stress and related genes during *C*Las infection.

## 5. Conclusions

This study identified three endoplasmic reticulum (ER) stress-related genes, *BiP*, *IRE1*, and *XBP1*, in *Diaphorina citri* and analyzed their spatiotemporal expression profiles. The research further characterized a key indicator of ER stress–*XBP1* splicing—and predicted its occurrence in other insect species. A semi-quantitative RT-PCR method was established to detect ER stress in *D. citri*. During *C*Las infection, ER stress exhibited a dynamic pattern of early induction followed by later suppression. Induction of ER stress using thapsigargin increased *C*Las titers in *D. citri*. RNA interference experiments revealed that silencing *IRE1* elevated *C*Las titers, whereas silencing *XBP1* reduced them. Together, these findings indicate that the IRE1-XBP1 pathway plays an important role in modulating *C*Las infection in *D. citri*.

## Data Availability

Data will be made available on request.

## Supplementary Materials

The following supporting information can be downloaded at: https://www.mdpi.com/article/10.3390/insects17030260/s1, Figure S1: Sequence alignment of BiP; Figure S2: Phylogenetic analysis of BiP and IRE1; Figure S3: Sequence alignment of IRE1; Figure S4: Amplification and phylogenetic analysis of *XBP1*; Figure S5: Predicted 3D model of XBP1 and sequence alignment of the bZip domain; Figure S6: Specificity assessment of qPCR primers designed for quantifying *XBP1* splicing. Table S1: Primers used in this study; Table S2: Bioinformatics of three ER stress-associated genes of *D. citri*; Table S3: Sequence identities of BiP protein across different pests and mammals; Table S4: Sequence identities of IRE1 protein across different pests and mammals; Table S5: Sequence identities of XBP1-U protein across different pests and mammals; Table S6. dsRNA sequences used in this study.
