# Supplementary material for: Molecular Characterization of Endoplasmic Reticulum (ER) Stress-Associated BiP, IRE1, and XBP1 Genes in Diaphorina citri and Their Roles During Candidatus Liberibacter asiaticus Infection
Source: Insects. 2026 Feb 28;17(3):260. doi: 10.3390/insects17030260 (PMC13027231; doi:10.3390/insects17030260)
Supplement: Supplementary file 1 [file insects-17-00260-s001.zip › Fig S6-Specificity assessment of qPCR primers designed for quantifying XBP1 splicing.pdf]

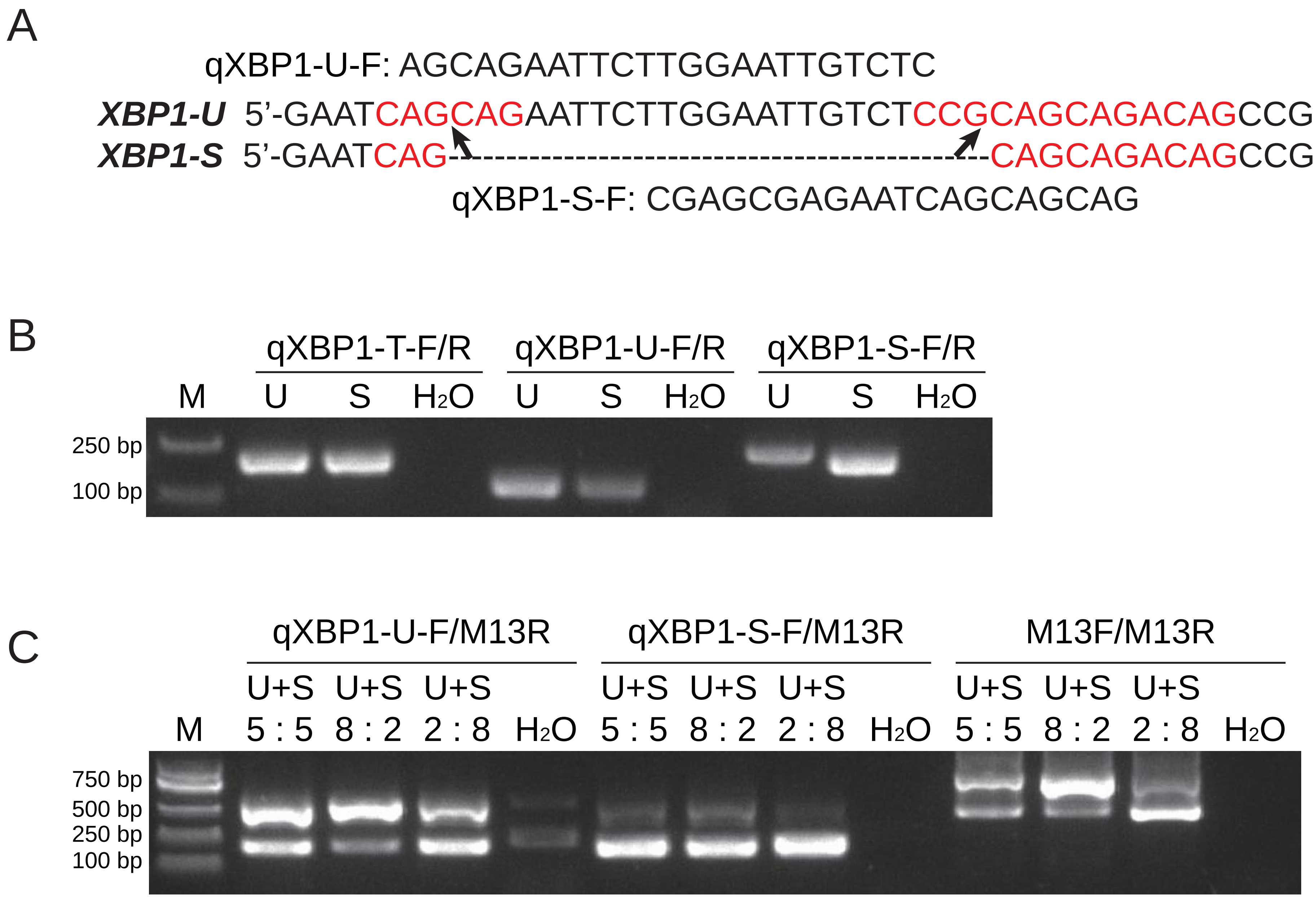
A
qXBP1-U-F: AGCAGAATTCTTGGAATTGTCTC
XBP1-U 5'-GAATCAGCAGAATTCTTGGAATTGTCTCCGCAGCAGACAGCCGG-3'
XBP1-S 5'-GAATCAG--CAGCAGACAGCCGG-3'
qXBP1-S-F: CGAGCGAGAATCAGCAGCAG
B
qXBP1-T-F/R
qXBP1-U-F/R
qXBP1-S-F/R
M U S H2O U S H2O U S H2O
250 bp
100 bp
C
qXBP1-U-F/M13R
qXBP1-S-F/M13R
M13F/M13R
U+S 5 : 5
U+S 8 : 2
U+S 2 : 8
H2O
M
750 bp
500 bp
250 bp
100 bp
