# Supplementary material for: Molecular Characterization of Endoplasmic Reticulum (ER) Stress-Associated BiP, IRE1, and XBP1 Genes in Diaphorina citri and Their Roles During Candidatus Liberibacter asiaticus Infection
Source: Insects. 2026 Feb 28;17(3):260. doi: 10.3390/insects17030260 (PMC13027231; doi:10.3390/insects17030260)
Supplement: Supplementary file 1 [file insects-17-00260-s001.zip › Supplementary figure legends.pdf]

Fig S1-Sequence alignment of BiP

(A) Full-length multiple sequence alignment of BiP orthologs from various pests and mammals.

(B, C) Enlarged views showing the alignment of the N-terminal signal peptide (B) and the C-terminal ER retrieval signal (KDEL motif and its variants) (C), respectively. Sequence conservation is visualized by color intensity (red, high conservation; blue, low conservation).

Fig S2-Phylogenetic analysis of BiP and IRE1

Maximum likelihood phylogenetic trees of BiP (Left) and IRE1 (Right) orthologs from diverse species. The *D. citri* sequence is marked in red with a pentagram. Trees were constructed using the Muscle alignment algorithm and the Maximum Likelihood method in MEGA software, with branch support assessed by 1000 bootstrap replicates.

Fig S3-Sequence alignment of IRE1

(A) Full-length multiple sequence alignment of IRE1 orthologs from various pests and mammals.

(B) Enlarged views of the sequence alignment flanking the conserved phosphorylated Ser site. Sequence conservation is visualized by color intensity (red, high conservation; blue, low conservation).

Fig S4-Amplification and phylogenetic analysis of *XBP1*

(A) Full-length amplification of *XBP1* transcripts.

(B) Detection and validation of the *XBP1* splicing event by EcoRI digestion. PCR products containing the unspliced *XBP1-U* or spliced *XBP1-S* were either untreated (upper panel) or digested with EcoRI (lower panel). The enzyme specifically cleaves *XBP1-U* (due to an intron-retained EcoRI site) but not XBP1-S, thereby clearly distinguishing the two isoforms.

(C) Maximum likelihood phylogenetic trees of XBP1 orthologs from diverse species. The *D. citri* sequence is marked in red with a pentagram. Trees were constructed using the Muscle alignment algorithm and the Maximum Likelihood method in MEGA software, with branch support assessed by 1000 bootstrap replicates.

Fig S5-Predicted 3D model of XBP1 and sequence alignment of the bZip domain

(A) Predicted three-dimensional structures of the unspliced XBP1-U and spliced XBP1-S protein isoforms. The conserved bZIP domains are highlighted, and model quality scores (pLDDT: predicted Local Distance Difference Test; pTM: predicted Template Modeling score) are provided.

(B) Multiple sequence alignment of the bZIP domain from XBP1 orthologs of various insect pests and mammals. Sequence conservation is visualized by color intensity (red, high conservation; blue, low conservation).

Fig S6-Specificity assessment of qPCR primers designed for quantifying *XBP1* splicing.

(A) Schematic of the *XBP1* splicing region, highlighting the CAG repeats near the splice site that complicate specific primer design.

(B) Specificity test using individual plasmid templates. PCR was performed with plasmids containing either the unspliced (*XBP1-U*) or spliced (*XBP1-S*) sequence, using primer sets specific for *XBP1-S*, *XBP1-U*, or total *XBP1* (*XBP1-T*, control). Both splicing-isoform-specific primer sets generated non-specific amplicons.

(C) Specificity test under mixed-template conditions. A plasmid containing a longer *XBP1-U* fragment was mixed with the *XBP1-S* plasmid at varying ratios prior to amplification. A faint band in the water control lane is likely due to PCR contamination but does not affect the main conclusion that specific detection of *XBP1-S* remains challenging.
