# Supplementary figures and images for: Molecular Characterization of Endoplasmic Reticulum (ER) Stress-Associated BiP, IRE1, and XBP1 Genes in Diaphorina citri and Their Roles During Candidatus Liberibacter asiaticus Infection
Source: Insects. 2026 Feb 28;17(3):260. doi: 10.3390/insects17030260 (PMC13027231; doi:10.3390/insects17030260)

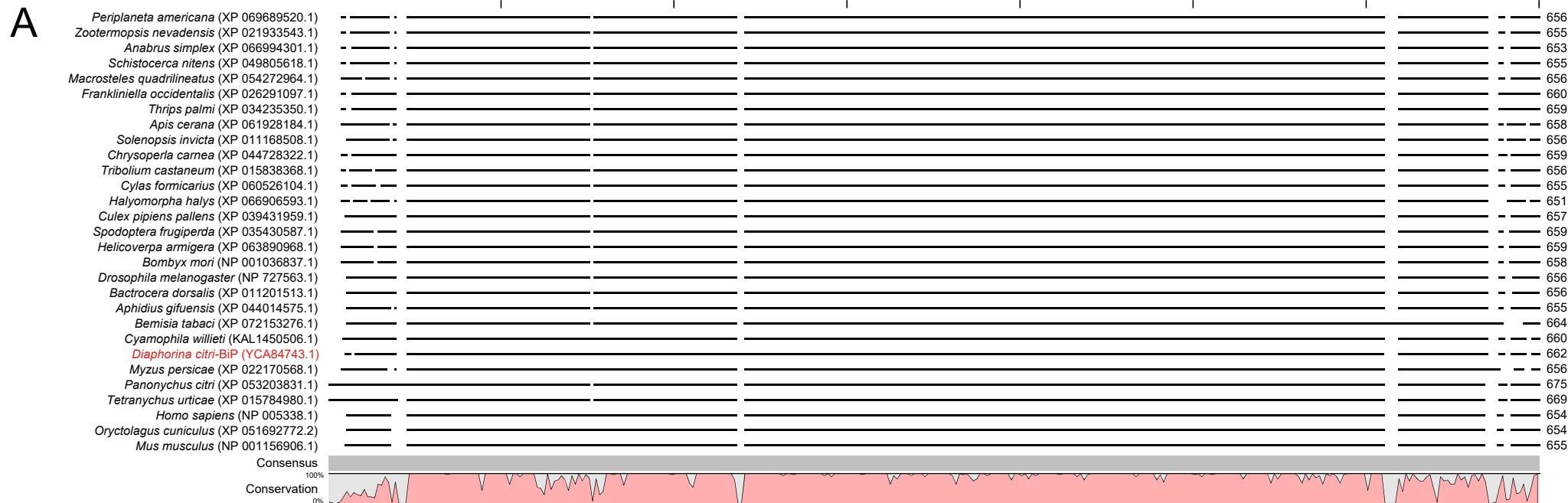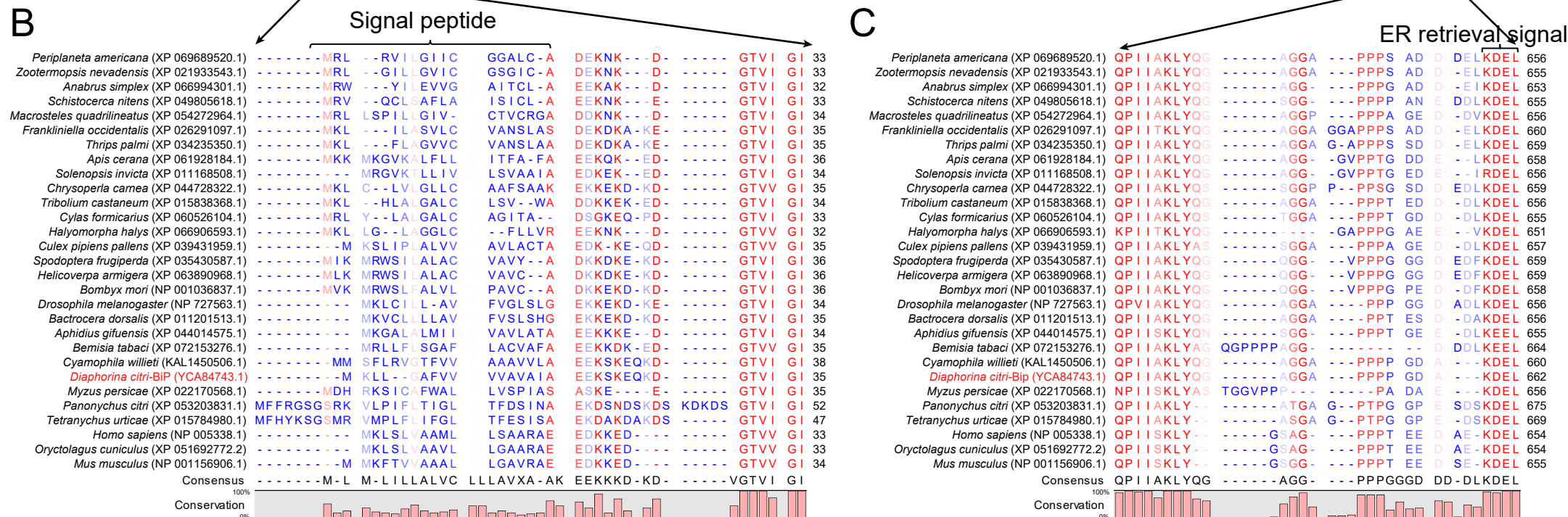

Supplement: Supplementary file 1 [file insects-17-00260-s001.zip › Fig S1-Sequence alignment of BiP.pdf]

A

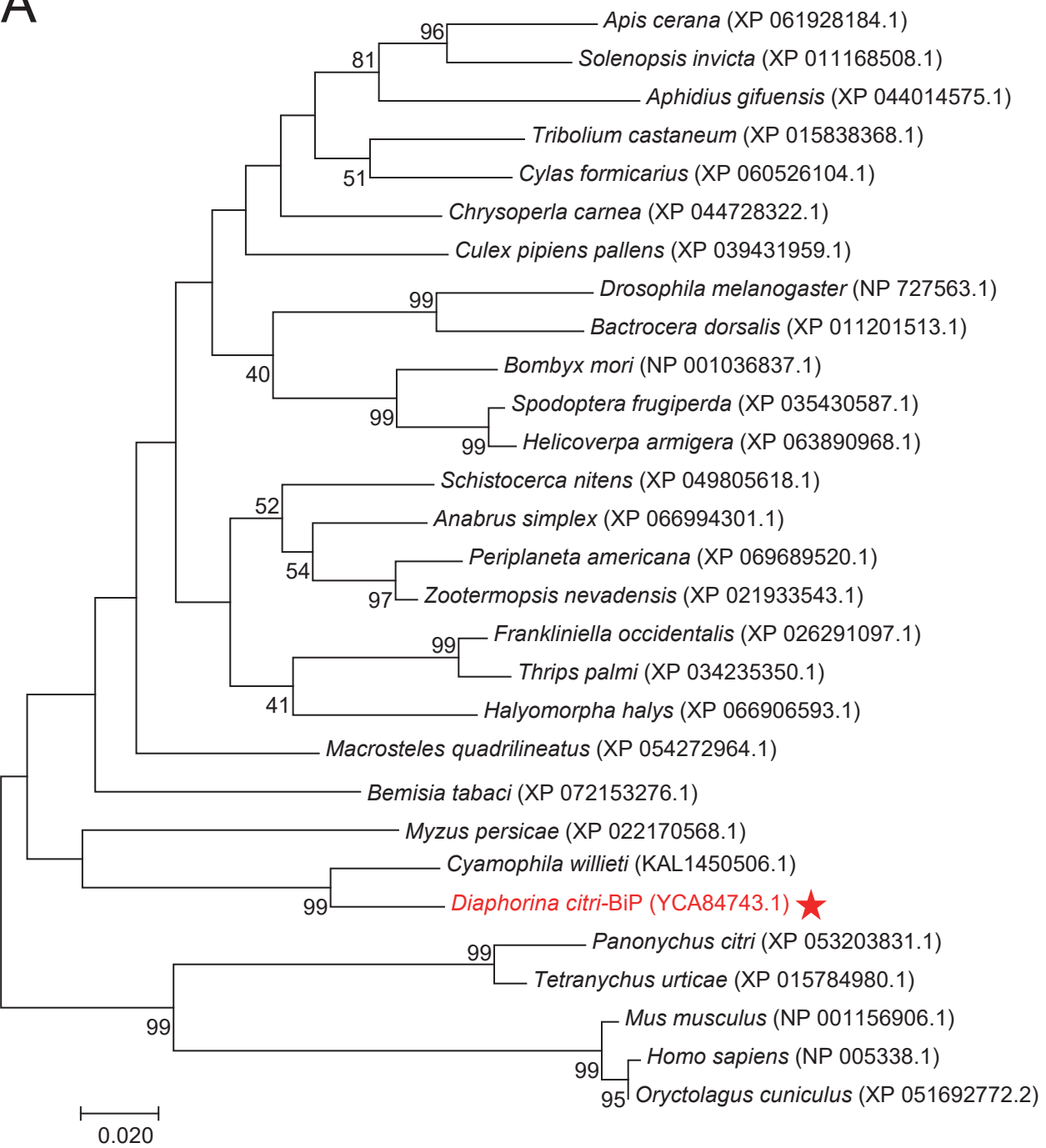

B

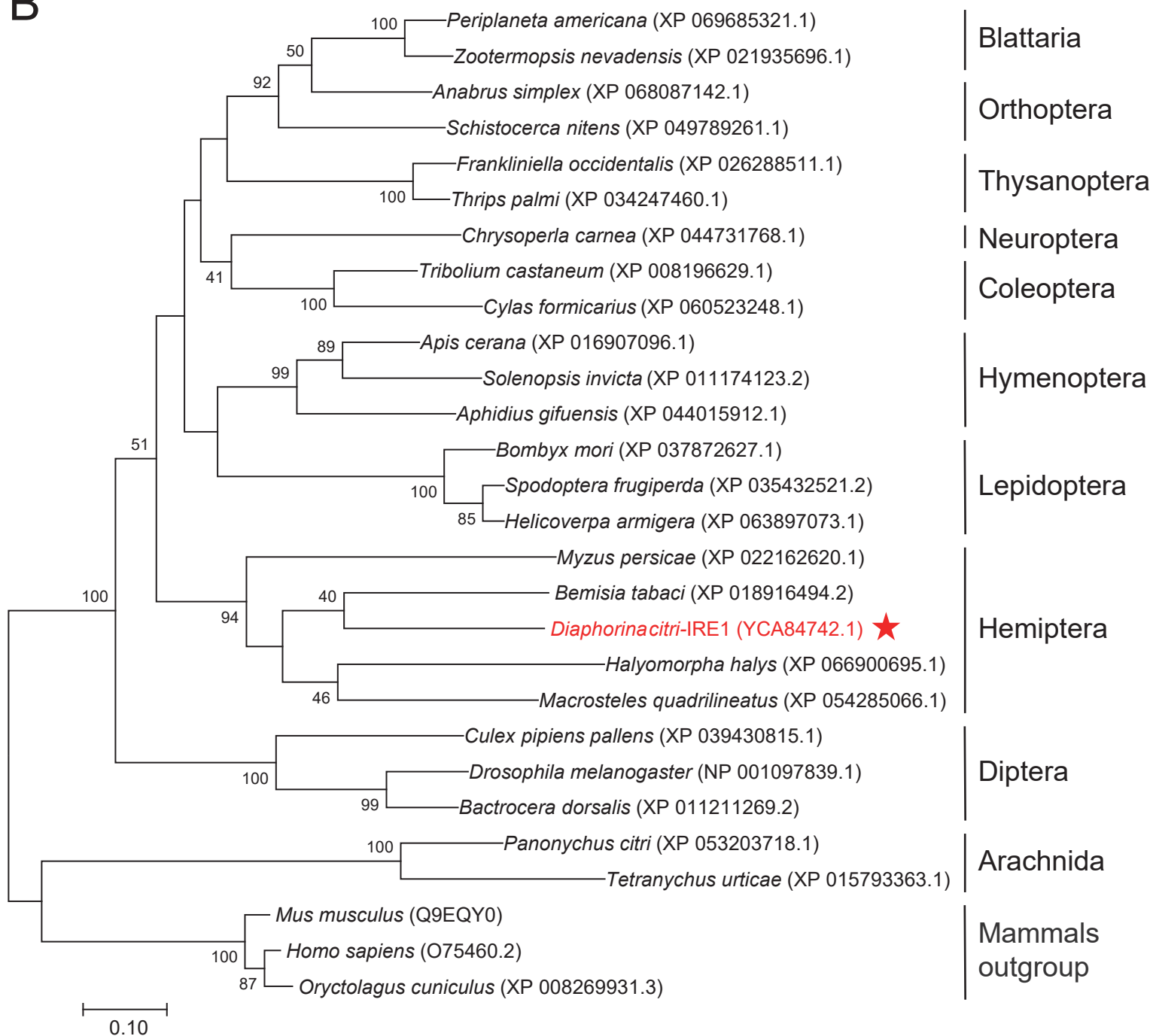

Supplement: Supplementary file 1 [file insects-17-00260-s001.zip › Fig S2-Phylogenetic analysis of BiP and IRE1.pdf]

A

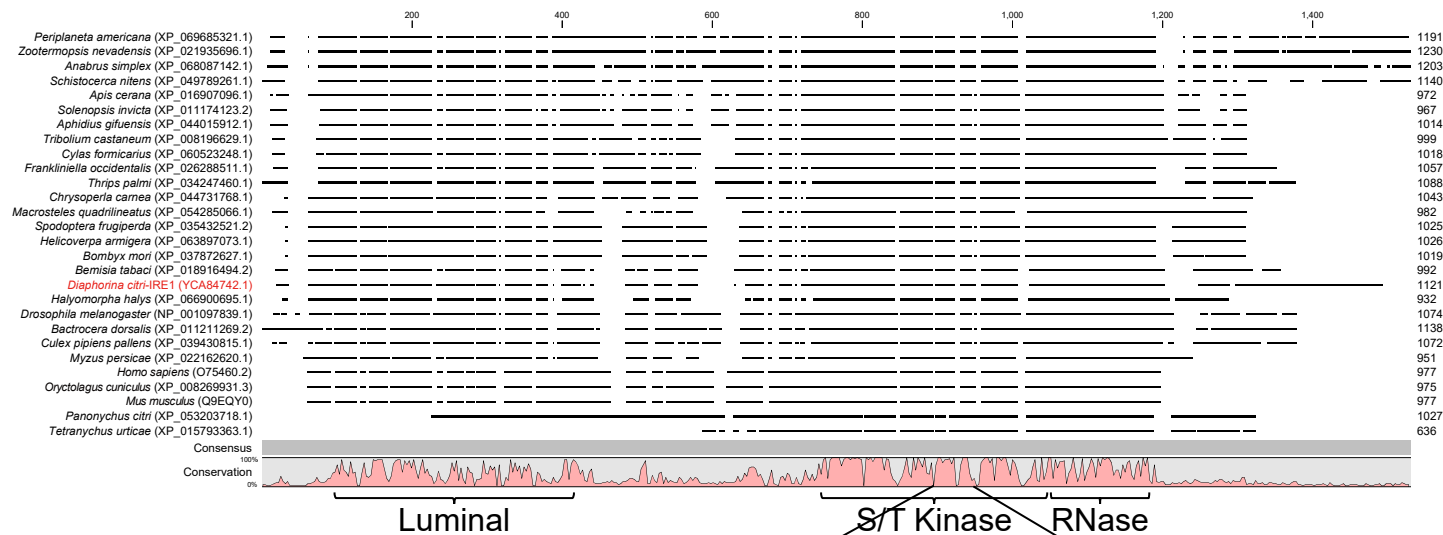

B

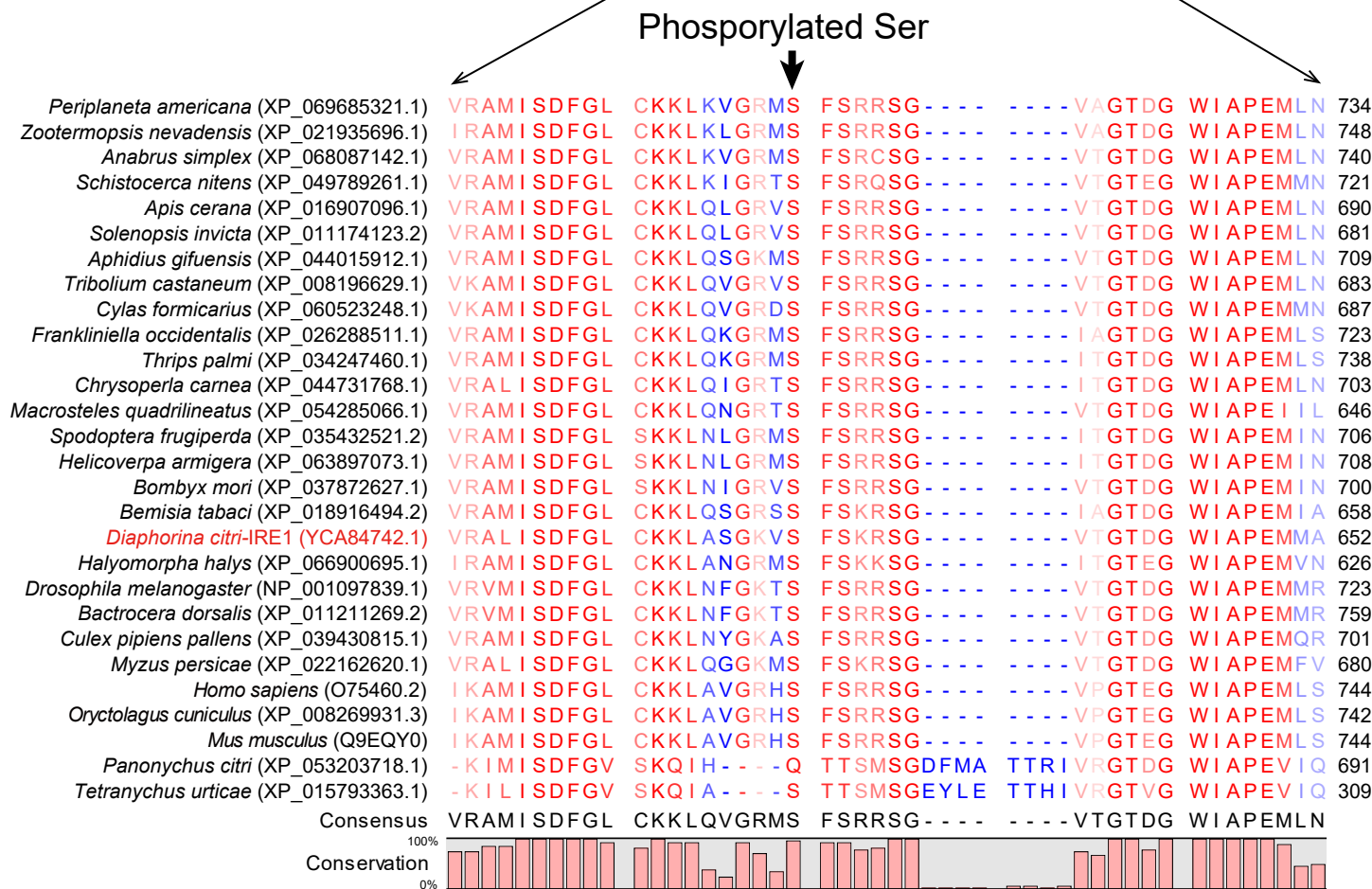

Supplement: Supplementary file 1 [file insects-17-00260-s001.zip › Fig S3-Sequence alignment of IRE1.pdf]

**A**

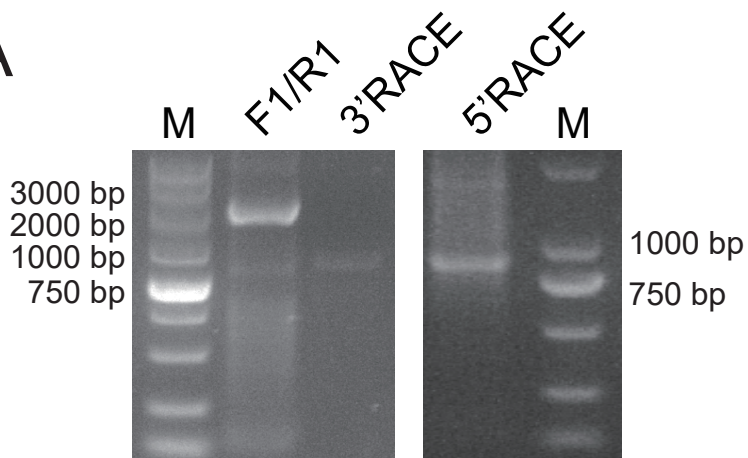

**B**

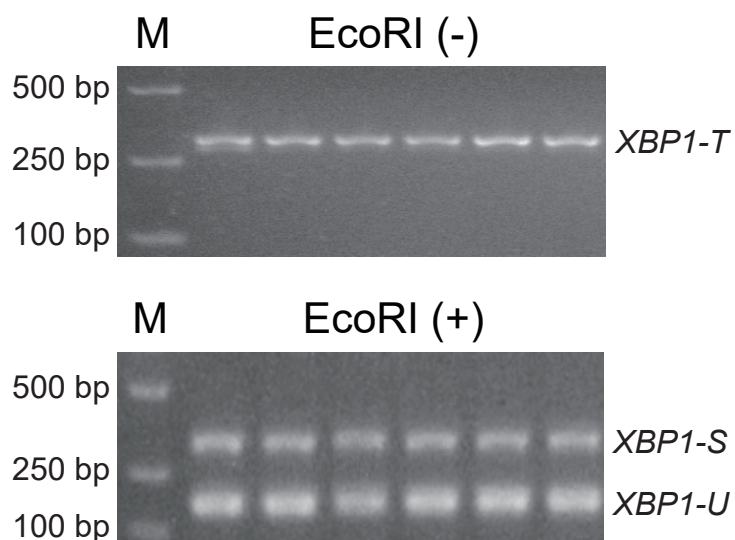

**C**

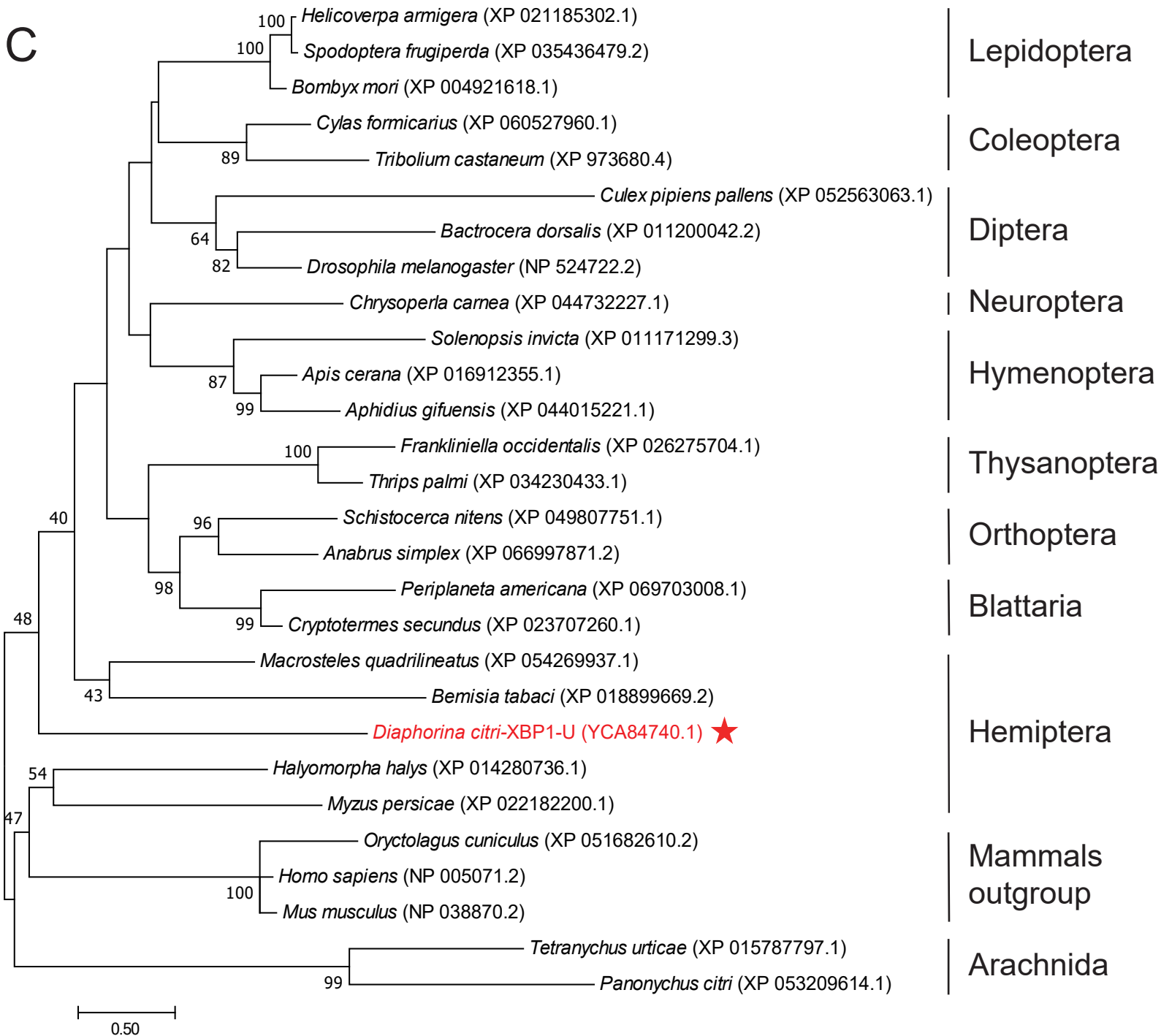

Supplement: Supplementary file 1 [file insects-17-00260-s001.zip › Fig S4-Amplification and phylogenetic analysis of XBP1.pdf]
