# Supplementary material for: Molecular Characterization of Endoplasmic Reticulum (ER) Stress-Associated BiP, IRE1, and XBP1 Genes in Diaphorina citri and Their Roles During Candidatus Liberibacter asiaticus Infection
Source: Insects. 2026 Feb 28;17(3):260. doi: 10.3390/insects17030260 (PMC13027231; doi:10.3390/insects17030260)
Supplement: Supplementary file 1 [file Fig S5-Predicted 3D model of XBP1 and sequence alignment of the bZip domain.pdf › Fig S5-Predicted 3D model of XBP1 and sequence alignment of the bZip domain.pdf]

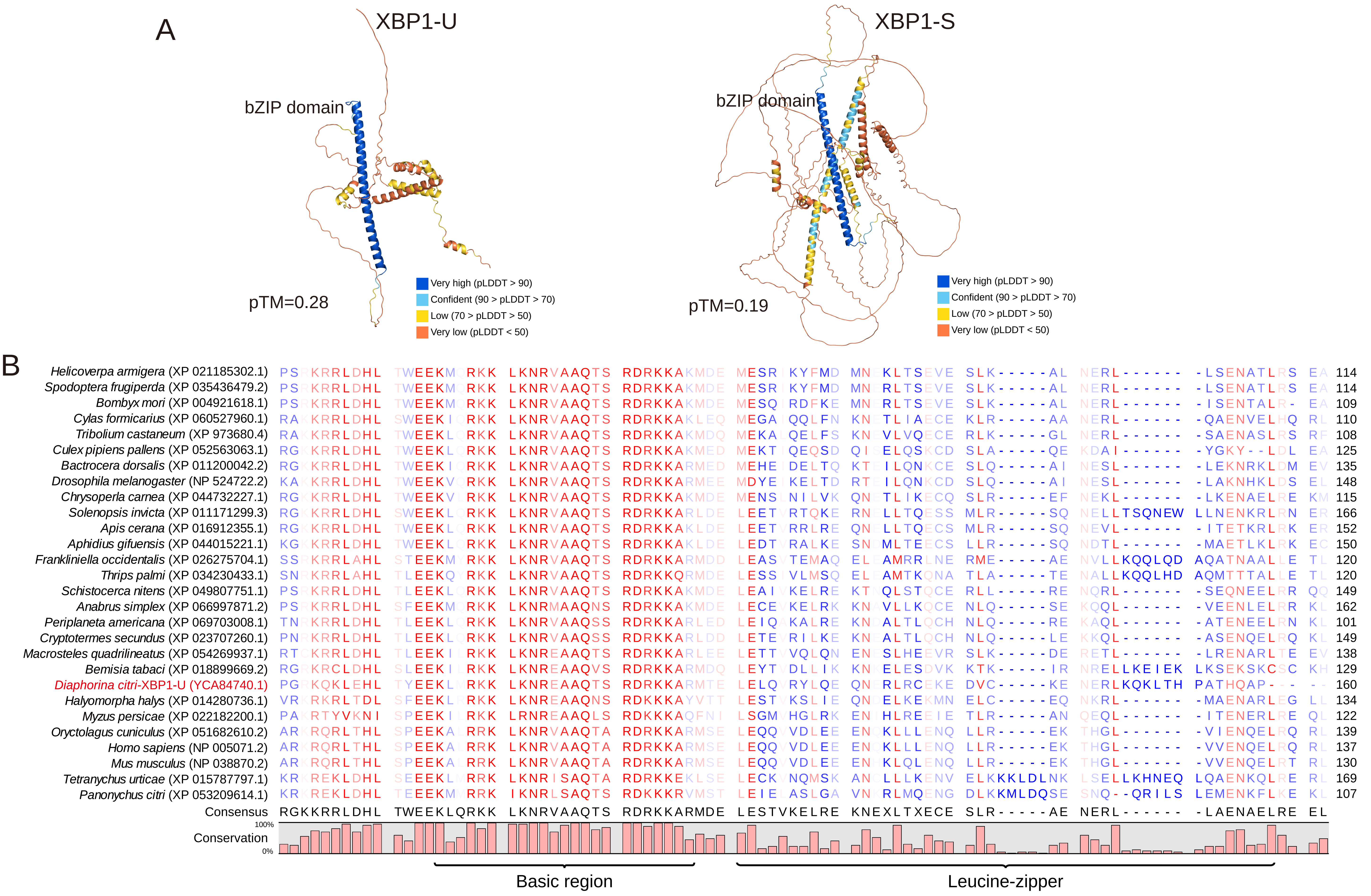

A
XBP1-U
bZIP domain
pTM=0.28
Very high (pLDDT > 90)
Confident (90 > pLDDT > 70)
Low (70 > pLDDT > 50)
Very low (pLDDT < 50)
XBP1-S
bZIP domain
pTM=0.19
Very high (pLDDT > 90)
Confident (90 > pLDDT > 70)
Low (70 > pLDDT > 50)
Very low (pLDDT < 50)
B
Helicoverpa armigera (XP 021185302.1) 114
Spodoptera frugiperda (XP 035436479.2) 114
Bombyx mori (XP 004921618.1) 109
Cylas formicarius (XP 060527960.1) 110
Tribolium castaneum (XP 973680.4) 108
Culex pipiens pallens (XP 052563063.1) 125
Bactrocera dorsalis (XP 011200042.2) 135
Drosophila melanogaster (NP 524722.2) 148
Chrysoperla carnea (XP 044732227.1) 115
Solenopsis invicta (XP 011171299.3) 166
Apis cerana (XP 016912355.1) 152
Aphidius gifuensis (XP 044015221.1) 150
Frankliniella occidentalis (XP 026275704.1) 120
Thrips palmi (XP 034230433.1) 120
Schistocerca nitens (XP 049807751.1) 149
Anabrus simplex (XP 066997871.2) 162
Periplaneta americana (XP 069703008.1) 101
Cryptotermes secundus (XP 023707260.1) 149
Macrosteles quadrilineatus (XP 054269937.1) 138
Bemisia tabaci (XP 018899669.2) 129
Diaphorina citri-XBP1-U (YCA84740.1) 160
Halyomorpha halys (XP 014280736.1) 134
Myzus persicae (XP 022182200.1) 122
Oryctolagus cuniculus (XP 051682610.2) 139
Homo sapiens (NP 005071.2) 137
Mus musculus (NP 038870.2) 130
Tetranychus urticae (XP 015787797.1) 169
Panonychus citri (XP 053209614.1) 107
Consensus RGKKRRLDHL TWEEKLQRKK LKNRVAAQTS RDRKKARMDE LESTVKELRE KNEXLTXECE SLR-----AE NERL------ -LAENAELRE EL
100%
Conservation
0%
Basic region
Leucine-zipper
